# Identification, Synthesis, and In Vitro Activities of Antimicrobial Peptide from African Catfish against the Extended-Spectrum Beta-Lactamase (ESBL)-Producing *Escherichia coli*

**DOI:** 10.3390/pharmaceutics16070850

**Published:** 2024-06-22

**Authors:** Hedmon Okella, Steven Odongo, Didier Vertommen, Emmanuel Okello

**Affiliations:** 1 Veterinary Medicine Teaching and Research Center, School of Veterinary Medicine, University of California, Davis, CA 93274, USA; hokella@ucdavis.edu; 2 Department of Biotechnical and Diagnostic Sciences, College of Veterinary Medicine, Animal Resources and Biosecurity, Makerere University, Kampala P.O. Box 7062, Uganda; 3 de Duve Institute and MASSPROT Platform, UCLouvain, 1200 Brussels, Belgium; 4 Department of Population Health and Reproduction, School of Veterinary Medicine, University of California, Davis, CA 95616, USA

**Keywords:** antimicrobial peptides, catfish, drug discovery, ESBL, LC-MS/MS

## Abstract

The global surge in multi-drug resistant bacteria, including extended-spectrum β-lactamase (ESBL)-producing *Escherichia coli* has led to a growing need for new antibacterial compounds. Despite being promising, the potential of fish-derived antimicrobial peptides (AMPs) in combating ESBL-producing *E. coli* is largely unexplored. In this study, native African catfish antimicrobial peptides (NACAPs) were extracted from the skin mucus of farmed African catfish, *Clarias gariepinus*, using a combination of 10% acetic acid solvent hydrolysis, 5 kDa ultrafiltration, and C_18_ hydrophobic interaction chromatography. Peptides were then sequenced using Orbitrap Fusion Lumos Tribrid Mass Spectrometry. The identified peptides were screened for potential antibacterial activity using Random Forest and AdaBoost machine learning algorithms. The most promising peptide was chemically synthesized and evaluated in vitro for safety on rabbit red blood cells and activity against ESBL-producing *E. coli* (ATCC 35218) utilizing spot-on-lawn and broth dilution methods. Eight peptides ranging from 13 to 22 amino acids with molecular weights between 968.42 and 2434.11 Da were identified. Peptide NACAP-II was non-hemolytic to rabbit erythrocytes (*p* > 0.05) with a zone of inhibition (ZOI) of 22.7 ± 0.9 mm and a minimum inhibitory concentration (MIC) of 91.3 ± 1.2 μg/mL. The peptide is thus a candidate antibacterial compound with enormous potential applications in the pharmaceutical industry. However, further studies are still required to establish an upscale production strategy and optimize its activity and safety in vivo.

## 1. Introduction

Antimicrobial resistance (AMR) remains a critical global public health threat responsible for at least 1.27 million deaths and about 5 million associated deaths in 2019 [1]. According to the World Health Organization, bacterial strains capable of producing extended-spectrum β-lactamase (ESBL) enzymes are among strains of first (critical) priority for new antibiotics [2]. The ESBL enzyme hydrolyzes a broad spectrum of beta-lactam antibiotics, rendering such antibiotics worthless [3]. Production of ESBL enzymes has been reported in several bacteria, including *Escherichia coli*, *Klebsiella* spp., *Shigella sonnei*, *Proteus mirabilis*, *Serratia marcescens*, Citrobacter freundii, *Salmonella* spp., and *Acinetobacter* spp. [4]. In any case, ESBL-producing *E. coli* represents a major challenge [5]. The search for novel forms of antimicrobial agents against ESBL producing bacteria is an essential approach to combat the ESBL threats [6]. To this effect, the potentials of plant-derived compounds [7,8,9], nanoparticles [10,11], propolis derivatives [12], and bacteriophages [13,14] in combating ESBL producing bacteria have been reported.

The African catfish, *Clarias gariepinus* is a benthopelagic freshwater scale-less fish [15], and their skin mucus is endowed with antimicrobial peptides (AMPs) [16,17,18]. With a generally recognized as safe (GRAS) status [19], broad-spectrum [20,21], rapid action [22], short length [19] AMPs are considered a promising fountain of novel antimicrobial drug candidates. Farmed fish have been identified as a potential source of new drug candidates. Researchers have isolated several antimicrobial peptides from the skin mucus of farmed fish species, including Oncoryncin II from farmed rainbow trout (*Oncorhynchus mykiss)* [23], histone H2B-derived antimicrobial peptide from the mucus of farmed Atlantic cod, *Gadus morhua* [24], Myxinidin from farmed hagfish (*Myxine glutinosa)* [25], and recently, EMP-Ag-NPs from farmed African catfish (*Clarias gariepinus)* [26]. Despite these potentials, no published record exists on the structure and safety profiles of AMPs in farmed African catfish. Moreover, our previous studies solely focused on the structure and potency of mucosal AMPs from wild African catfish [16,17,18] leaving the need to compare such attributes, especially the antimicrobial activity of mucosal AMPs of farmed African fish. Thus, in the quest for potential drug leads against ESBL-producing bacteria, the current study presents findings on the structure, in vitro activities, and safety antimicrobial peptides in the skin mucus of farmed African catfish.

## 2. Materials and Methods

### 2.1. Fish and Mucus Collection

Live mature African catfish of both sexes (*n* = 8, weight range 210–398 g) were purposively sampled from a local farm in Seeta, Mukono, Central Uganda. The fish were washed in deionized water (pH 7.01), and skin mucus (20 mL) was harvested via dorso-lateral scrapping of the fish skin using a sterile plastic spatula. The ventral skin mucus was not collected to avoid sperm, anal, and intestinal contamination. The harvested skin mucus was immediately homogenized in an equal volume of 10% (*v*/*v*) acetic acid for 1 min using a polytron homogenizer (Kinematica, Malters, Switzerland) and incubated for 5 min in a water bath at 95 °C [25]. Insoluble mucus constituents were separated via centrifugation at 10,000× *g* at 4 °C for 1 h (Hermle, Wehingen, Germany), and the supernatant was obtained for further purification.

### 2.2. Peptide Purification

To remove the high-molecular-weight proteins, the supernatant (20 mL) was filtered through a 5 kDa molecular weight cut-off membrane (MWCO) (Sartorius, Gloucestershire, UK) via centrifugation at 6000× *g* for 10 h at 4 °C. Low-molecular-weight peptides (usually below 5 kDa) exhibit high mobility and a higher ligand efficiency to penetrate the bacterial cell membrane for intracellular targets [27]. The molecular cut-off process also eliminates high-molecular-weight hydrolytic enzymes that could potentially confound the activity of extracted peptides [28]. The ultra-filtrate was then purified via hydrophobic interaction chromatography utilizing C_18_ stationary phase cartridges (50 μm particle size, 60 Å pore diameter, and 15 mL column volume) containing 5 g sorbent (Thermo Scientific, Bellefonte, PA, USA). The column was conditioned by running 30% (*v*/*v*) acetonitrile at a flow rate of 1 mL/min prior to sample loading. The hydrophobic peptides bind to the hydrophobic matrix, while the contaminants flow through. Momentarily bound peptides were then washed with deionized water (pH 7.01), and, finally, eluted with 5 mL of 70% (*v*/*v*) acetonitrile [29]. To completely remove acetonitrile and other contaminants, the elute was lyophilized at −104 °C and 0.013 millibars (Labconco, Kansas, MI, USA). The concentration of the lyophilized peptides was determined using a Nanodrop spectrophotometer at 280 nm. Here, 1 µL of 1X PBS was loaded in an auto blank mode, followed by the lyophilized peptides in 1X PBS solvent. The absorbance of the peptides was then measured at 280 nm as a wavelength of maximum absorbance by aromatic rings on amino acids [30]. Samples were then shipped to de Duve Institute, UClouvain (Belgium) for peptide sequencing.

### 2.3. Peptide Sequencing

The detailed procedure for peptide sequencing has been previously described [18]. Briefly, we used the Orbitrap Fusion Lumos Tribrid Mass Spectrometer (Thermo Fisher, Waltham, MA, USA) to determine the molecular mass and the amino acid sequence of peptides. First, the freeze-dried sample was reconstituted at a concentration of 1 mg/mL in distilled water and then diluted 10-fold in a solution containing 3% acetonitrile (ACN) and 0.1% trifluoroacetic acid (TFA). Next, 2 μL of the peptides was loaded directly onto a reversed-phase pre-column (Acclaim PepMap 100, Thermo Scientific, Waltham, MA, USA) and eluted in backflush mode. The peptide separation process was carried out using a reversed-phase analytical column equilibrated in a solution containing 3.5% ACN and 0.1% formic acid (FA) in water (Acclaim PepMap RSLC, 0.075 mm × 250 mm, Thermo Scientific, Waltham, MA, USA) with a specific gradient of solvent B. This process was performed at a constant flow rate of 300 nL/min on the Ultimate 3000 RSLC nanoHPLC system (Thermo Fisher Scientific, Waltham, MA, USA). The peptides were then subjected to a Nanospray Ionization (NSI) source followed by tandem mass spectrometry (MS/MS) in the Orbitrap Fusion Lumos, which was coupled online to the nanoLC. Pseudo-molecular ions and detected in the Orbitrap at a resolution of 120,000 within a mass-charge (*m*/*z*) range from 350 to 1500. A charge state decision tree was applied prior to MS2 fragmentation. Ions with charge states +2 and +3 were selected for MS/MS using Higher-energy C-trap dissociation (HCD) at a setting of 30; ion fragments were detected in the Orbitrap at a resolution of 30,000. For precursors with charge states from +3 up to +8, MS/MS was obtained via Electron-Transfer/Higher-Energy Collision Dissociation (EThcD) fragmentation with supplemental energy set at 30, and ion fragments were detected in the Orbitrap at a resolution of 30,000. A data-dependent procedure of MS/MS scans was applied for the top precursor ions above a threshold ion count of 2.0 × 10^4^ in the MS survey scan with a dynamic exclusion of 60.0 s. The total cycle time was set to 4 s. The mass spectrometry proteomics data have been deposited to the ProteomeXchange Consortium via the PRIDE [31] partner repository with the dataset identifier PXD049239 and 10.6019/PXD049239. The resulting MS/MS data were processed using the Sequest HT search engine within Proteome Discoverer 2.4 against a custom database containing 102,688 sequences from catfish species (compiled from Uniprot proteins entries with taxonomy ID: 175774 *Bagarius yarrelli*; 310915 *Pangasianodon hypophtalmus*; 7998 *Ictalurus punctatus*; 35657 *Clarias microcephalus*; and 219545 *Ameiurus melas*). No enzyme was specified as the cleavage enzyme allowing a maximum peptide length of 30 residues, four modifications per peptide b- and y- ions for HCD fragmentation, and b- c- z- and y- ions for EThcD fragmentation. The mass error was set to 10 ppm for precursor ions and 0.1 Da for fragment ions. Oxidation on methionine (Met) was considered as variable modification. Peptide matches were filtered using the q-value and Posterior Error Probability calculated using the Percolator algorithm ensuring an estimated false positive rate (FDR) below 5%. The filtered Sequest HT output files for each peptide were grouped according to the protein from which they were derived.

### 2.4. Primary Structure Characterization

The physical and chemical parameters of the identified peptides were predicted using the Expert Protein Analysis System (ExPASy) proteomics tool at the Swiss Institute of Bioinformatics (https://web.expasy.org/protparam/ accessed on 26 September 2023). Later, guided by the physiochemical characteristics of the peptides, the Moon and Fleming scale [32] was utilized to predict the antibacterial potentials of the peptides at the Database of Antimicrobial Activity and Structure of Peptides (https://dbaasp.org/tools?page=linear-amp-prediction accessed on 26 September 2023). Random Forest and AdaBoost machine learning algorithms at the same web server were then utilized in microbial strain-specific predictions of AMPs incorporating reference standards (*Escherichia coli* ATCC 25922 and *Staphylococcus aureus* ATCC 25923) under default settings [33]. Here, putative antimicrobial peptides were predicted as having MIC < 25 μg/mL and non-active peptides as having MIC > 100 μg/mL.

### 2.5. The 3-D Structure Prediction of the Antimicrobial Peptide

The 3-D structures of the most promising putative antimicrobial peptides were modelled de novo utilizing PEP-FOLD V3.5 at https://bioserv.rpbs.univ-paris-diderot.fr/services/PEP-FOLD3/ accessed on 2 January 2024 [34]. FASTA files were used as input for 100 default simulations. Output models were ranked based on sOPEP energies and Apollo-predicted melting temperature. To validate the predicted structure, we submitted the same FASTA file sequences to the Iterative Threading Assembly Refinement (I-TASSER) server (https://zhanglab.dcmb.med.umich.edu/I-TASSER/ accessed on 2 January 2024) [35]. The server uses protein templates identified using the Local Meta-Threading Server (LOMETS) from the Protein Data Bank (PDB) library (https://www.rcsb.org/ accessed on 2 January 2024) to predict 3-D structures guided by their confidence score [36]. The quality of the modeled structures was then evaluated by estimating their stereochemical properties using a Ramachandran Plot Server (https://saves.mbi.ucla.edu/ accessed on 4 January 2024).

### 2.6. Peptide Synthesis

The NACAP-II (LANVLFRRNATTILQ) peptide was synthesized in a 9-fluorenyl methoxycarbonyl (Fmoc) solid-phase method by GenScript^®^ Corporation (Piscataway, NJ, USA). Synthesized NACAP-II peptide was purified via high-performance liquid chromatography (HPLC) using an Inertsil ODS-SP 4.6 mm × 250 mm. As per the manufacturers protocols, a linear gradient from solvent A [0.065% Trifluoroacetic acid (TFA) in water] to solvent B (0.05% TFA in Acetonitrile) at a flow rate of 1.0 mL/min and run-time of 25 min was utilized. The peptides were then detected at 220 nm through UV spectrophotometry and the characterized mass spectrometer positive mode of an ESI source. Synthesized peptide was resuspended as per manufacturers recommendations and its concentration determined using a NanoDrop One Spectrophotometer (Thermo Fisher, Waltham, MA, USA).

### 2.7. Antimicrobial Activity

#### 2.7.1. Spot-on-Lawn Method

The spot-on-lawn assay was conducted as previously described by Soomro and colleagues [37] with minor modifications. Here, 100 µL of ESBL-producing *E. coli* ATCC 35218 (106 CFU/mL in PBS) were spread-plated on Muller Hinton Agar (MHA) as a control organism recommended by the Clinical and Laboratory Standards Institute (CLSI) [38]. Twenty-five µL of laboratory-prepared NACAP-II peptides were spotted onto the overlaid surface and the plates were left to dry for 1 h. Ciprofloxacin (25 μg/mL) was spotted as the positive control drug as a recommended concentration for agar-based susceptibility tests, while 1X PBS was used as negative control. The plates were incubated at 37 °C for 18 h and were subsequently examined for zone of inhibition.

#### 2.7.2. Broth Dilution Method

The broth dilution method was performed as previously described [39], with minor modifications using ESBL-producing *E. coli* ATCC 35218 as the test organism. Briefly, 100 μL of Tryptic Soy Broth (TSB) was added into each well of a 96-well plate (Dynatec, El Paso, TX, USA). An equal volume of the 1 mg/mL peptide solution NACAP-II was then added to the first well with TSB, and the two-fold serial dilution was then prepared by transferring 100 μL of the mixture to an equal volume of TSB in the next well, up to the 8th dilution and 100 μL of the mixture was discarded from the eighth well. The two-fold serial dilutions of the positive control (50 μg/mL ciprofloxacin) (Cyman, Ann Arbor, MI, USA) and the negative control (TSB) were similarly prepared. The concentration of Ciprofloxacin was doubled to 50 μg/mL to accommodate the two-fold dilution up to the 8th well. Thereafter, 5 μL of the bacterial suspension (1.5 × 10^6^ cells/mL) was added to all test wells, mixed thoroughly, and incubated overnight incubation at 37 °C. Subsequently, 5 μL of 6.75 mg/mL Resazurin (Thermo Scientific, Ward Hill, MA, USA) was added to all wells and the plate was incubated for another 4 h at 37 °C, and color changes were physically observed and recorded. Microbial growth was indicated by an irreversible color change from blue resazurin to pink resorufin. The lowest concentration without color change was considered the minimum inhibitory concentration (MIC). All dilutions (test and negative and positive controls) were performed in triplicate.

### 2.8. Hemotoxicity Profile of Antimicrobial Peptide

Hemolytic activity was assayed with a modified red blood cell method described by Bea and others [40]. Rabbit red blood cells were purchased from Innovative Research Inc. (Novi, MI, USA). The blood was washed by resuspending in three volumes of 1X PBS, and the suspension was centrifuged at 1000× *g* for 10 min at 4 °C, and the supernatant was discarded. The process was repeated 2 more times. The sedimented cells were resuspended in an equal volume of 1X PBS and stored at 4 °C until use. To measure hemolytic activity, aliquots of the peptide’s solution were added to 100 mL of 1% rabbit erythrocytes in 1X PBS, in 2 mL centrifuge tubes, to final concentrations of 1, 10, 50, and 100 µg/mL. The cell suspensions were incubated at 37 °C for 1 h. The non-lysed erythrocytes were removed via centrifugation (16,000× *g*) and the released hemoglobin was determined spectrophotometrically at 414 nm using a NanoDrop One Spectrophotometer (Thermo Fisher, Waltham, MA, USA). The control for no release of hemoglobin was a sample of 1% erythrocytes incubated in 1X PBS without peptide. The control for 100% release of hemoglobin was a sample of 1% erythrocyte incubated in PBS with 1% TritonX-100. Each assay was performed for three independent experiments, and data were expressed as the mean and SEM. The percentage of hemolysis was calculated using the following formula:

Hemolysis (%) = [(A_e_ − A_n_)/(A_p_ − A_n_)] × 100%,

where A_e_ is absorbance of the peptide, A_p_ is the absorbance of the positive control, and A_n_ is the absorbance of the negative control.

## 3. Results

### 3.1. Solid Phase Extraction

After allowing the contaminants to flow through the C_18_ column, followed by a deep washing of the bound-peptide matrix, the concentration of the hydrophobic peptides in the lyophilized elute was found to be 69.12 mg/mL.

### 3.2. Peptide Identification and Characterization

**Figure 1:**
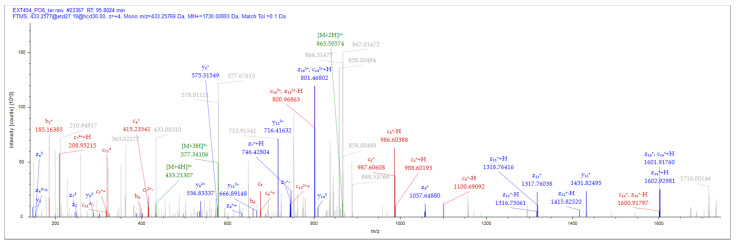
NACAP-II peptide mass spectrum. The representative MS2 data were sourced out of +4 parent ion. A 433.257 m/z utilized EThcD fragmentation and Orbitrap detection with resolution at 30.000. Peptide, LANVLFRRNATTILQ from protein, W5UKU1 was identified using a series of ions (y-, b-, c-, and z-).

A total of eight (8) putative antimicrobial peptides were identified by matching the tandem mass spectral data of peptides with amino acid sequences in the local catfish database using Sequest HT. All the eight peptides were short in length of 13–22 amino acid residues with a net charge range of −4 to + 2 (Table 1) at an isoelectric point ranging from 3.77 to 12.00. Most of these peptides (62.5%) had a more positive grand average of hydropathicity (GRAVY) implying they are more hydrophobic, with NACAP-VI demonstrating the highest GRAVY of 0.431 followed by NACAP-II (0.373). Two of the identified peptides were predicted to be antimicrobial. Upon subjecting the two predicted antimicrobial peptides to a more rigorous strain-specific antimicrobial peptide prediction, only one LC-MS/MS identified peptide (Figure 1) was predicted to be active against the selected strains.

### 3.3. The 3-D Structure Modelling

**Figure 2:**
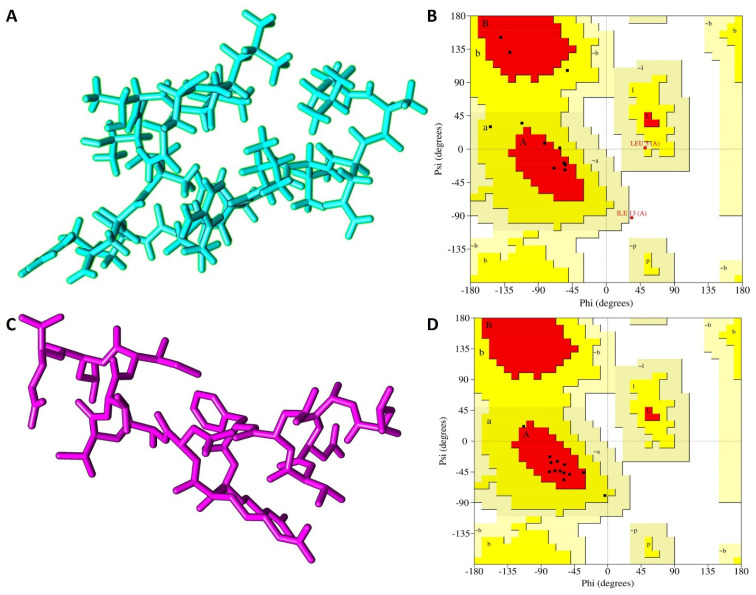
Predicted peptide 3-D structures of NACAP-II together with respective Ramachandran validation plots. (**A**) NACAP-II modeled using I-TASSER in stick format. (**B**) NACAP-II Ramachandran plot modeled using I-TASSER. (**C**) NACAP-II modeled using PEP-FOLD. (**D**) NACAP-II Ramachandran plot modeled using PEP-FOLD. Red regions (A, B and L) represent locations of residues in most favored region; Yellow regions (a, b, and l) represent locations of residues in additional allowed regions; Citrine regions (~a, ~b, ~l and ~p) represents locations of residues in generously allowed regions; White regions represents locations of residues in disallowed regions. Most of the amino acid residues in the PEP-FOLD-modeled NACAP-II peptide were in the most favored region (84.6%), with no residue in the disallowed region.

Both the I-TASSER and PEP-FOLD predictions yielded five models for peptide NACAP-II. To identify the best, I-TASSER model, a standard −5 to 2 confidence score (c-score) scale was applied. The I-TASSER model_1 showed the highest c-score of −1.21, signifying the model had the best prediction confidence and was thus selected as the best I-TASSER model. For the PEP-FOLD prediction, the model that releases the highest amount of sOPEP energy at the highest melting temperature was selected as the best model. Here, PEP-FOLD model_1 released the highest amount of energy (−19.47) and highest melting temperature (0.53) and was considered for the downstream analysis (Figure 2). Upon structural evaluation utilizing the Ramachandran plot, the PEP-FOLD modeled 3-D structure was found to be of higher quality compared to the I-TASSER model. A majority of the amino acid residues (84.6%) of the PEP-FOLD modeled NACAP-II 3-D structure were in the most favored region (Table 2), compared to the I-TASSER modeled 3-D structure (53.8%). There was no residue in the PEP-FOLD modeled 3-D structure in the disallowed region.

### 3.4. Peptide Synthesis and In Vitro Antimicrobial Activity

**Figure 3:**
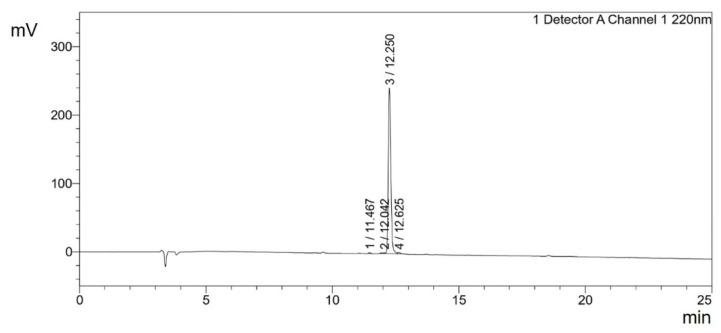
Chromatogram of the NACAP-II peptide in the 25 min run at 220 nm. Inertsil ODS-SP 4.6 mm × 250 mm reversed-phase high-performance liquid chromatography (RP-HPLC) column with the gradient from solvent A [0.065% Trifluoroacetic acid (TFA) in water] to solvent B (0.05% TFA in Acetonitrile) at a flow rate of 1.0 mL/min.

**Figure 4:**
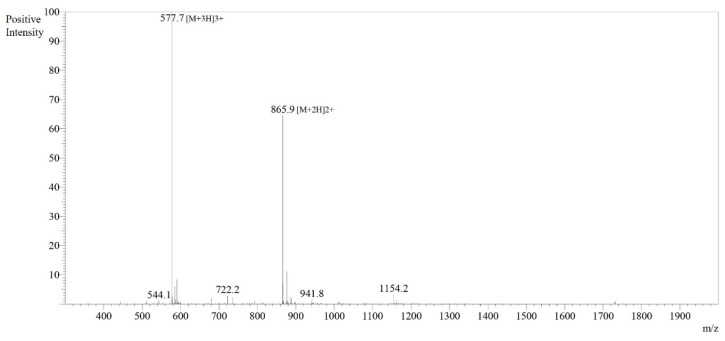
Mass spectrum for peptide NACAP-II. The mass spectra for peptide NACAP-II were obtained using a 50% H_2_O/50% MeOH water dissolution method prior to a 0.1 µL injection. Electrospray Ionization (ESI) interface was utilized at +4.5 kV detector. Observed mass was 1730.01, while the theoretical mass was 1730.03.

**Figure 5:**
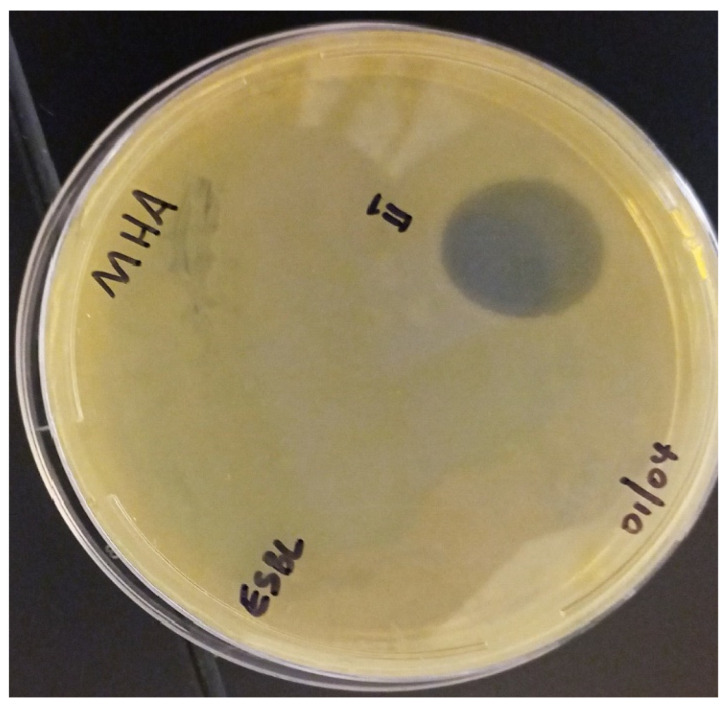
Zone of inhibition (ZOI) for NACAP-II peptide on ESBL-producing *E. coli*. NACAP-II peptide was active with a zone of inhibition of 22.7 ± 0.9 mm. The ESBL-producing *E. coli* was cultured on Mueller-Hinton Agar (MHA). The experiment was performed in triplicate, and data were expressed as the mean and SEM.

The synthesized peptides were detected at the wavelength of 220 nm to a purity of 97.4% (Figure 3). The mass spectrum (Figure 4) of the synthesized peptides indicated that their molecular mass was the same as that established during peptide sequencing (1730.1 Da). The synthesized peptide NACAP-II showed a 22.7 ± 0.9 mm zone of inhibition (Figure 5) and a 91.3 ± 1.2 μg/mL minimum inhibitory concentration against ESBL-producing *E. coli*.

### 3.5. Hemolytic Activity of the Antimicrobial Peptide

**Figure 6:**
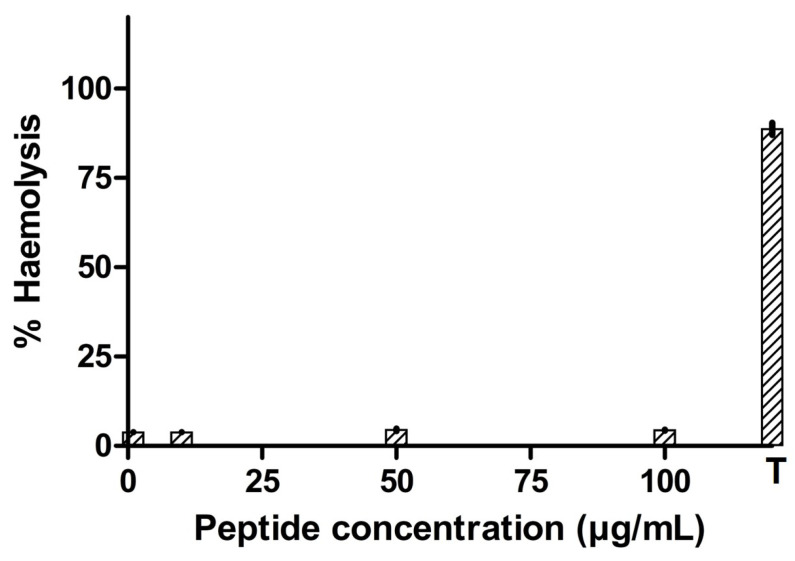
T-1% Triton X-100. Percentage of hemolysis of rabbit red blood cells via synthesized antimicrobial peptide. The concentration of (1–100) µg/mL did not hemolyze the rabbit blood cells. The experiment was performed in triplicate, and mean values were reported.

After incubation of the synthesized peptide in rabbit erythrocytes, the percentage of hemolysis generally increased with an increase in the peptide concentration (Figure 6). But, for the tested concentrations, peptide NACAP-II was found not to be hemolytic (*p* = 0.198) at the test value of the blank. This generally signifies the potential of peptide NACAP-II being safe on mammalian red blood cells.

## 4. Discussion

The study results demonstrated that peptides extracted from the skin mucus of farmed *C. griepinus* are active against one of the WHO’s first (critical) priority pathogens, ESBL-producing *E. coli* (MIC: 91.3 ± 1.2 μg/mL), with a promising in vitro safety profile. The peptide extraction processes involved both the ultrafiltration membrane and C_18_ hydrophobic interaction chromatography, in which ultrafiltration isolated peptides of lower molecular weights and desired functional properties [41], while the solid-phase hydrophobic matrix optimally captured hydrophobic peptides [29].

When compared to the activity of earlier reported fish mucosal AMPs on other bacterial strains, NACAP-II demonstrated a lower MIC compared to ACAP-IV (MIC 520.7 μg/mL) from the wild African catfish, *Clarias garienpinus* [17]. However, its value was slightly higher than that a Goldenstriped soapfish-derived peptide Gs D (FIGGIISFFKRLF) (MIC, 13.3–50 μg/mL) [42] and that of Pelteobagrin (GKLNLFLSRLEILKLFVGAL) sourced from the yellow catfish, *Pelteobagrus fulvidraco* (MIC, 2–64 μg/mL) [43]. Kęska and Stadnik [44] attributed such variation in the antimicrobial activity of the peptide to physicochemical properties of the peptide including charge, molecular size, structure, and hydrophobicity. A net positive charge is vital for the potency of AMPs [45,46], by enhancing the peptide’s affinity for negatively charged phospholipids on the outer surface of the microbial cell membrane through electrostatic interactions [47]. On par with earlier reported fish-derived mucosal AMPs like Myxinidin [25] and peptide ACAP-V [18], peptide NACAP-II was cationic (+2), possibly justifying its action on ESBL-producing *E. coli*. On the other hand, the modeled structure NACAP-II peptide was found to be less stable when compared to the ACAP-IV peptide from wild African catfish that had all its residues (100.0%) for both I-TASSER and PEP-FOLD in the most favored regions [17].

Hydrophobic peptides, on the other hand, readily interact with the hydrophobic lipid bilayer to permeate bacterial cell [20,48]. With the abundance of hydrophobic residues such as Alanine, Valine, Isoleucine, Glycine, Isoleucine, Leucine, Phenylalanine, Proline, and Tryptophan, peptide NACAP-II showed a higher GRAVY (0.373) compared to the earlier reported in peptide A15_B [49]. This may additionally contribute to its effectiveness against ESBL-producing *E. coli*, given that the NACAP-II peptide can easily disrupt bacterial cell membranes, leading to cell lysis and death.

Like other mucosal AMPs from the Yellow catfish, *Pelteobagrus fulvidraco* [43]; Topmouth culter, *Erythroculter ilishaeformis* [50]; Atlantic salmon, *Salmo salar* [51]; and Winter flounder, *Pleuronectes americanus* [52], peptide NACAP-II had a low LC-MS/MS determined molecular weight (1730.01 Da). Peptides of lower molecular weight exhibit higher mobility when crossing the periplasm [27]. This study believes this may, in addition, explain the relatively low MIC demonstrated by peptide NACAP-II on extended-spectrum beta-lactamases producing *E. coli*.

As another parameter in drug discovery, toxicity remains one of the key drawbacks [53]. Up to the maximum tested concentration of 100 μg/mL, peptide NACAP-II was found to be non-hemolytic to the rabbit erythrocytes, implying it can possibly stay on the surface of the mammalian cell membrane without penetrating it [54]. Similar findings of non-toxicity have been reported in Moronecidin-like peptide from *Hippocampus comes* [55] and angiotensin-converting enzyme inhibitory peptides sourced out of *Oncorhynchus mykiss* [56]. Thus, with this promising level of safety, the activity of peptide NACAP-II can potentially be optimized against extended-spectrum beta-lactamases producing *E. coli*.

## 5. Conclusions

This study identified peptide NACAP-II, LANVLFRRNATTILQ, as the most promising peptide against extended-spectrum beta-lactamases producing *E. coli* with an MIC of 91.3 ± 1.2 μg/mL and a non-hemolytic effect on the rabbit red blood cells. The peptide is thus a candidate antibacterial compound with enormous potential applications in the pharmaceutical industry. However, further studies are still required to establish its mode of action, upscaled production strategy, and optimized activity and safety in vivo.

## Figures and Tables

**Table 1:** Physiochemical properties of native African catfish antimicrobial peptides (NACAPs).

Peptide ID	Sequence	Length	MW (Da)	Calculated Net Charge	pI	GRAVY	General Antimicrobial Activity
NACAP-I	VAPEEHPVLLTEAPLNPK	18	1954.06	−2	4.75	−0.267	Non-AMP
NACAP-II	LANVLFRRNATTILQ	15	1730.01	2	12.00	0.373	AMP
NACAP-III	TQAADLLGMVSRKSLWGQF	19	2108.09	1	8.41	0.053	Non-AMP
NACAP-IV	MLNLLHIKSGGCFLFLE	17	1951.02	0	6.50	1.047	Non-AMP
NACAP-V	KGVQINYRYFKGFFLES	17	2096.20	2	9.52	−0.359	Non-AMP
NACAP-VI	GAGGGFGAGGGFG	13	968.42	0	5.22	0.431	AMP
NACAP-VII	NDDLIPPAQSIETTDMKTEVNC	22	2434.11	−4	3.77	−0.673	Non-AMP
NACAP-VIII	VSTMKSFMPGPCIHGDLP	18	1948.90	0	6.71	0.172	Non-AMP

Peptide ID, Peptide Identity; GRAVY, Grand average of hydropathicity; AMP, Active antimicrobial peptide; non-AMP, non-antimicrobial peptide; pI, Isoelectric point.

**Table 2:** Evaluation scores for the predicted 3-D peptide structures.

Ramachandran Plot Parameters	I-TASSER	PEP-FOLD
Residues in most favored regions (%)	53.8	84.6
Residues in additional allowed regions (%)	30.8	15.4
Residues in generously allowed regions (%)	7.7	0.0
Residues in disallowed regions (%)	7.7	0.0

## Data Availability

The mass spectrometry proteomics data can be downloaded at: https://www.ebi.ac.uk/pride/ deposited on 7 February 2024 with the dataset identifier PXD049239 and 10.6019/PXD049239.
